# Association of a SNP in *SLC35F3* Gene with the Risk of Hypertension in a Chinese Han Population

**DOI:** 10.3389/fgene.2016.00108

**Published:** 2016-06-20

**Authors:** Xiao-Li Zang, Wei-Qing Han, Feng-Ping Yang, Kai-Da Ji, Ji-Guang Wang, Ping-Jin Gao, Guang He, Sheng-Nan Wu

**Affiliations:** ^1^Shanghai Key Laboratory of Vascular Biology, Ruijin Hospital, Shanghai Jiao Tong University School of MedicineShanghai, China; ^2^Shanghai Institute of Hypertension, Shanghai Jiao Tong University School of MedicineShanghai, China; ^3^Laboratory of Vascular Biology, Institute of Health Sciences, Shanghai Institutes for Biological Sciences, Chinese Academy of SciencesShanghai, China; ^4^State Key Laboratory of Medical Genomics, Shanghai Jiao Tong UniversityShanghai, China; ^5^Key Laboratory for the Genetics of Developmental and Neuropsychiatric Disorders (Ministry of Education), Bio-X Institutes, Shanghai Jiao Tong UniversityShanghai, China

**Keywords:** *SLC35F3*, association, hypertension, susceptibility, SNP

## Abstract

A recent study suggested that *SLC35F3* which encoded a thiamine transporter was a new candidate gene for hypertension. The goal of this study was to investigate the association between the single-nucleotide polymorphisms (SNPs) in the *SLC35F3* gene and hypertension in a Chinese population. Sanger sequencing was performed in 93 samples to find SNPs in coding regions and intron–exon boundaries in the *SLC35F3* gene. We found eight genetic variants in the coding regions of *SLC35F3* and subsequently genotyped a non-synonymous variant rs34032258 (C > G) in 1060 hypertension patients and 1467 controls. After adjusting for age and gender, multivariate analysis of covariance showed that the variant was associated with hypertensive traits. In detail, diastolic blood pressure (DBP) was 8 mmHg higher, blood urea nitrogen was 12 mmol/L higher, and creatinine was 15 mmol/L lower in G/G group compared with C/C group (*p* = 0.007; 0.012 and 0.029, respectively). Further study suggested that C/G+G/G had higher DBP than C/C genotype in those whose DBP ≥ 90 mmHg (98.02 mmHg vs. 94.04 mmHg, *p* = 0.021). No significant difference has been found in systolic blood pressure between different genotypes. Additionally, in the subgroup of obesity, allele distribution of this variant has shown significant difference between hypertensive patients and normotensive controls (*p* = 0.018). In conclusion, we found that the rs34032258 in the *SLC35F3* gene was associated with high blood pressure and may increase the risk of hypertension. The new hypertension-susceptibility locus may involve in the pathogenesis of hypertension and indicate some novel treatment implications.

## Introduction

The prevalence of hypertension is increasing in most countries and hypertension is an important risk factor for the development of cardiac-cerebral vascular diseases. However, the cause still remains largely enigmatic (Evans et al., 2003; D’Agostino et al., 2008). Growing evidence showed that genetic and environmental factors played a crucial role in the onset of hypertension (Zhang et al., 2010; Munroe et al., 2011). According to single pressure value, the hypertensive heritability was 31–34%. In addition, average value from more than three measurements showed a higher heritability of 56–57% and it could reach as high as 63–68% based on 24 h-ambulatory blood ^1^pressure monitory (Kupper et al., 2006). The heritable trait remains the most potent and crucial risk factor for cardiovascular diseases, although details of its genetic determination are poorly understood (Munroe et al., 2011).

The solute carrier (SLC) group of transporters transports organic or inorganic molecules across cell or organelle membranes (Ishida et al., 2005; Zhang et al., 2012). Nearly 400 *SLC* members are organized into 52 families (Ishida and Kawakita, 2004). Members of the human solute carrier 35 (*SLC35*) transporter family, which encode for nucleotide sugar transporters, have been divided into six subfamilies (A–F; Ishida and Kawakita, 2004; Saier et al., 2015)and are predominately expressed in the lumen of the endoplasmic reticulum (ER) and the Golgi apparatus (Goda et al., 2006). Genetic mutations in *SLC35* transporter family have been found associated with cardiovascular diseases. In Zhang et al. (2014) have found the *SLC35F3* was associated with blood pressure in North America and Western Europe. *SLC35F3* sequence homolog to a putative yeast thiamine (vitamin B1) transporter is located at 1q42.2 with 9 exons. The *SLC35F3* mRNA was expressed at the highest levels in the adult cerebellum (Nishimura et al., 2008). Up to now, the effect of *SLC35F3* genetic variants on blood pressure has not been studied in Chinese populations. In this study, we explored whether variants in the coding regions of the *SLC35F3* gene contributed to blood pressure variation and hypertension.

## Materials and Methods

### Subjects

We conducted a two-stage strategy in this study. First, we scanned all the exons of *SLC35F3* gene in 93 hypertensive patients by Sanger sequencing. Subsequently, the most suspicious variants were further genotyped by TaqMan-MGB assays in a total of 2527 participants, including 1060 hypertensive patients and 1467 normotensive controls. All of the participants were examined at Shanghai Ruijin Hospital. Every participant signed a consent form, and the study was approved by the hospital’s ethics committee.

Age, gender, and medication usage were obtained from Shanghai Institute of Hypertension, Ruijin hospital. Height and weight were measured and BMI (kg/m^2^) was calculated. Blood pressure was measured using a calibrated mercury sphygmomanometer with appropriate adult cuff size by well-trained examiners. Diagnosis of hypertension was based on a mean SBP ≥ 140 mmHg and/or DBP ≥ 90 mmHg on two occasions, and/or current usage of anti-hypertensive treatment.

During different analytic processes, the samples selected were diverse and the details are showed in Supplementary Table S1. The characteristics of all the 2527 samples are showed in **Table 1**. All the hypertensive patients (*n* = 1060) were selected in order to analyze the effect of the SNP on hypertensive traits (**Table 2**). Based on the results in **Table 2**, we selected 578 patients whose DBP ≥ 90 mmHg to further verify the relationship between the SNP and DBP (**Figure 2**). In addition, allelic frequencies of rs34032258 in different BMI levels were analyzed between 1467 controls and 1035 cases excluding 25 unavailable BMI data in case groups (**Table 3**). The effect of gender on SBP and DBP is shown in **Table 4** including all the participants. Besides, the plasma of 344 hypertensive patients was available for ELISA test in order to verify the effect of this gene on blood thiamine in patients (**Figure 3**).

**Table 1:** General characteristics of the study population.

Variables	Controls (*n* = 1467)	Cases (*n* = 1060)	*p*
Gender (F/M)	764/703	522/538	0.160
Age (years)	63.11 @ 0.21	63.44 @ 0.16	0.236
Height (cm)	160.45 @ 0.20	162.59 @ 0.24	<0.001
Weight (kg)	58.87 @ 0.23	67.26 @ 0.31	<0.001
BMI (kg/m^2^)	22.85 @ 0.07	25.36 @ 0.09	<0.001
WC (cm)	80.26 @ 0.22	88.86 @ 0.26	<0.001
Hip (cm)	91.19 @ 0.15	96.31 @ 0.21	<0.001
SBP (mmHg)	115.50 @ 0.25	152.93 @ 0.57	<0.001
DBP (mmHg)	72.99 @ 0.18	91.41 @ 0.37	<0.001
TC (mmol/L)	4.85 @ 0.04	5.06 @ 0.11	0.031
TG (mmol/L)	1.46 @ 0.02	2.07 @ 0.19	0.002
BUN (mmol/L)	10.07 @ 0.44	11.62 @ 0.76	0.017
Cr (mmol/L)	70.18 @ 0.57	74.38 @ 1.03	<0.001

*BMI, body mass index; DBP, diastolic blood pressure; SBP, systolic blood pressure; WC, waist circumference; BUN, blood urea uitrogen; F, female; M, male; Cr, creatinine; TC, total cholesterol; TG, triglyceride. Statistically significant (p < 0.05).*

**Table 2:** *SLC35F3* variant rs34032258: effect on hypertensive traits in case group.

Genotype	SBP (mmHg)	DBP (mmHg)	BUN (mmol/L)	Cr (mmol/L)	BMI (kg/m^2^)
CC (*n* = 991)	152.85 ± 0.60	91.26 ± 0.38	11.04 ± 0.78	74.47 ± 1.01	25.38 ± 0.10
CG (*n* = 48)	158.00 ± 2.55	93.70 ± 1.91	8.33 ± 0.85	79.22 ± 3.51	25.24 ± 0.46
GG (*n* = 21)	157.86 ± 4.09	99.14 ± 2.71	23.73 ± 7.38	59.59 ± 7.78	24.41 ± 0.55
*p*	0.096	0.007^∗^	0.012^∗^	0.029^∗^	0.391

Effect of SLC35F3 variant rs34032258 on systolic blood pressure (SBP),diastolic blood pressure(DBP), blood urea nitrogen(BUN), Cr and body mass index(BMI) in case group (n = 1060). Analysis of covariance (ANCOVA), adjusted for age and gender.*Statistically significant (p < 0.05).

**Table 3:** Allelic frequencies of rs34032258 in different BMI levels.

	Groups	Allele		OR	*p*
		C	G		
Underweight	Cases (*n* = 8)	16 (1.000)	0 (0.000)		0.291
	Controls (*n* = 107)	200 (0.935)	14 (0.065)		
Normal	Cases (*n* = 228)	433 (0.949)	23 (0.051)	0.799	0.375
	Controls (*n* = 688)	1320 (0.959)	56 (0.041)		
Overweight	Cases (*n* = 245)	470 (0.959)	20 (0.041)	0.762	0.393
	Controls (*n* = 334)	647 (0.969)	21 (0.031)		
Obese	Cases (*n* = 554)	1061 (0.958)	47 (0.042)	1.344	0.018^∗^
	Controls (*n* = 338)	638 (0.944)	38 (0.056)		

BMI was layered according to local related guide in 2010 released by China health department. Underweight: BMI < 18.5; normal: 18.5 ≤ BMI<23; overweight: 23 ≤ BMI<25; obese: BMI ≥ 25. Case: n = 1035; control: n = 1467; Missed data in case was 25.*Statistically significant (p < 0.05).

**Table 4:** The effect of gender on SBP and DBP.

	Gender	SBP (mmHg)	*p*	DBP (mmHg)	*p*
Total	Male	131.22 ± 0.65	0.863	82.83 ± 0.39	<0.000^∗^
	Female	131.37 ± 0.68		78.78 ± 0.36	
Cases	Male	152.35 ± 0.76	0.145	93.57 ± 0.52	<0.000^∗^
	Female	154.04 ± 0.87		89.43 ± 0.52	
Controls	Male	115.91 ± 0.35	0.089	74.62 ± 0.25	<0.000^∗^
	Female	115.05 ± 0.35		71.50 ± 0.27	

One-way ANOVA was used to analyze between BP and sex in different groups. *Statistically significant (p < 0.05).

### DNA Samples

DNA was extracted from peripheral venous blood by lyzing red blood cells (RBCs) and digesting the remaining white cell pellet with proteinase K in accordance with the protocol (TIANamp Genomic DNA kit, Tiangen, China). DNA samples were stored at –80°C until additional analysis was finished.

### Selection and Genotyping of *SLC35F3* Polymorphisms

Sanger sequencing of 9 exons of the *SLC35F3* gene in 93 people was performed by Mapbioo Technology Company of China. Variant calling was carried out using Sequencher 5.1 and the annotation of the detected variants was checked on National Center for Biotechnology Information. We selected missense variant and designed primers for further genotyping.

Genotypes were determined by pre-designed TaqMan Allele Discrimination Assay (Cat. #4351379, primer forward, 5′-AGCGTGCGTCACTGAATGA-3′; reverse, 5′-ACACCCCCATGACTCAAGTG-3′, Life Technologies, USA). TaqMan polymerase chain reaction (PCR) was performed on a 7900 Real-Time PCR system (Applied Biosystems, Foster City, CA, USA) in total volume of 2.5 μL consisting of 2.4 μL TaqMan PCR Master Mix (Life Technologies, USA) and 0.03 μL genomic DNA. Cycling conditions were 95°C for 30 s, and 50 cycles of 95°C for 15 s, and 60°C for 1 min.

### Measurement of Thiamine B1

Thiamine B1 concentration was determined by using ELISA kit (E-EL-0007c, Elabscience).

### Statistical Analysis

Continuous variables expressed as mean ± standard error (SE) were compared between two groups by unpaired *t*-test. Relations between categorical variables were examined by χ^2^ test. The association of examined SNPs with hypertension as a binary trait and BP as a continuous trait was done by Logistic and linear regression analyses, respectively, after adjusting for age, gender, and BMI. MANCOVA was used to compare the differences of BP, BMI, BUN, and Cr across the genotypes of rs34032258 after treating age and gender as covariates. The frequencies of genotypes between patients and controls were estimated by SHE^2^. Considering the impact of antihypertensive regimens, SBP and DBP were added by a fixed value of 10 and 5 mmHg, respectively, according to a previous report (Cui et al., 2003). BMI was classified according to the guidelines released by the Ministry of Health of China in 2010. Statistical analyses were performed with SPSS version 13.0 for Windows. Two-sided *p* < 0.05 was considered to be significant.

## Results

### rs34032258 Missense Variant Detected in 93 Samples by Sanger Sequencing

**FIGURE 1:**
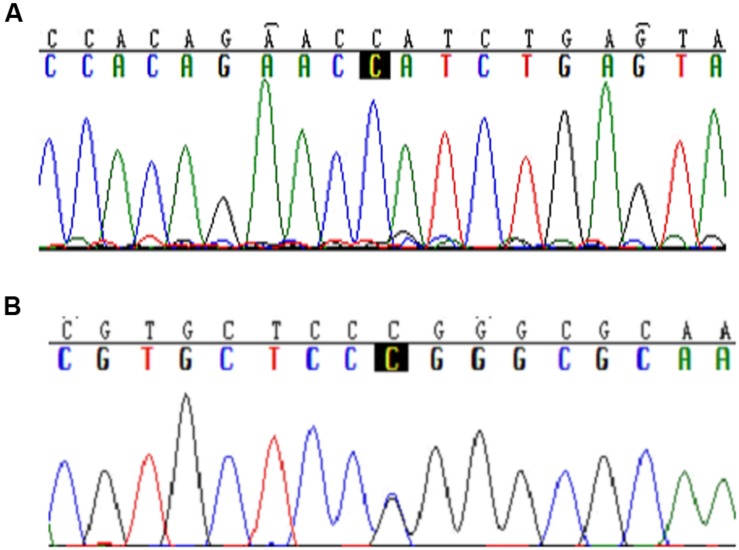
**Typical sequences of specific mutations in the *SLC35F3* SNPs in hypertensive patients. (A)** Rs16842784 was not found mutation in Chinese people. **(B)** C > G mutation in rs34032258.

In 93 hypertension patients, eight genetic variants were found in the coding regions of *SLC35F3*. Among them, a missense variant, rs34032258, was detected with an allele frequency of 3.73% (**Figure 1B**, 466C > G). However, SNPs previously reported to be associated with hypertension, either rs16842784 or rs17514104 was not found in the 93 samples (**Figure 1A**).

### Baseline Characteristics

The comparisons of the demographic and clinical features between hypertensive patients and normotensive controls are shown in **Table 1**. The distributions of age (*p* = 0.16) and gender (*p* = 0.24) did not differ significantly between patients and controls. BMI (*p* < 0.001), blood pressures (both *p* < 0.001) and Cr were significantly higher in patients than in controls as expected.

### Association of rs34032258 with Hypertension in Han Chinese

**FIGURE 2:**
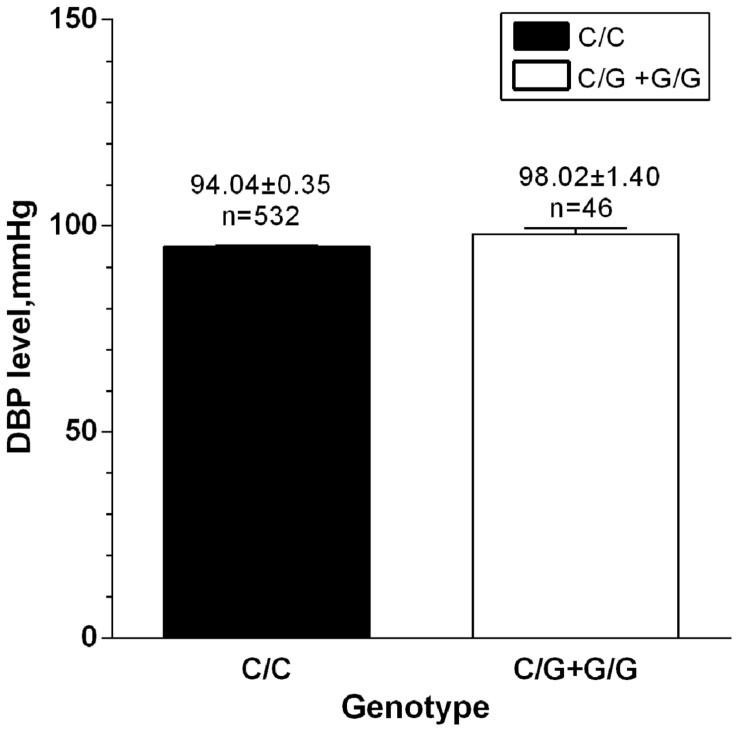
**DBP level between G-carriers and wild type in those whose DBP ≥ 90 mmHg.** *n* = 578, *p* = 0.021.

The associations of rs34032258 with BP, BMI, BUN, and Cr are shown in **Table 2**. After adjusting for age and gender, the result from MANCOVA showed that rs34032258 was associated with DBP, BUN, and Cr. In detail, DBP was 8 mmHg higher, BUN was 12 mmol/L higher, and Cr was 15 mmol/L lower in G/G group compared with C/C group. In contrast, there was no difference in SBP and BMI between the two groups. Further study suggested that C/G+G/G had higher DBP than C/C genotype in those whose DBP ≥ 90 mmHg (98.02 mmHg vs. 94.04 mmHg, *p* = 0.021; **Figure 2**). Notably, we found that G allele frequency was significantly lower in hypertensive patients compared with controls in obesity group (OR = 1.34, *p* = 0.018; **Table 3**). No significant difference was found between controls and cases in underweight, normal weight, or overweight subjects.

We also performed logistic regression analysis to assess the association between hypertension and rs34032258 after adjusting confounding factors such as age, gender, and BMI. The result exhibited that compared to C/C genotype, C/G and G/G genotypes were not statistically associated with the risk of hypertension, with the corresponding odds ratio of being 1.18 (95% CI: 0.64–2.12) and 1.25 (95% CI: 0.60–2.52), respectively (both *p* > 0.05). Moreover, we examined the association of rs34032258 with SBP and DBP on a continuous scale by using linear regression analysis with age, gender, and BMI as covariates, and as expected, this variant was significantly associated with SBP and DBP (*p* = 0.033 and 0.010), especially for the latter (data not shown).

To discover the relation between sex and BP, one-way ANOVA analysis was performed and the result showed that male had higher DBP both in case group (93.57 mmHg vs. 89.43 mmHg, *p* < 0.000; **Table 4**) and in control group (74.62 mmHg vs. 71.50 mmHg, *p* < 0.000; **Table 4**).

**FIGURE 3:**
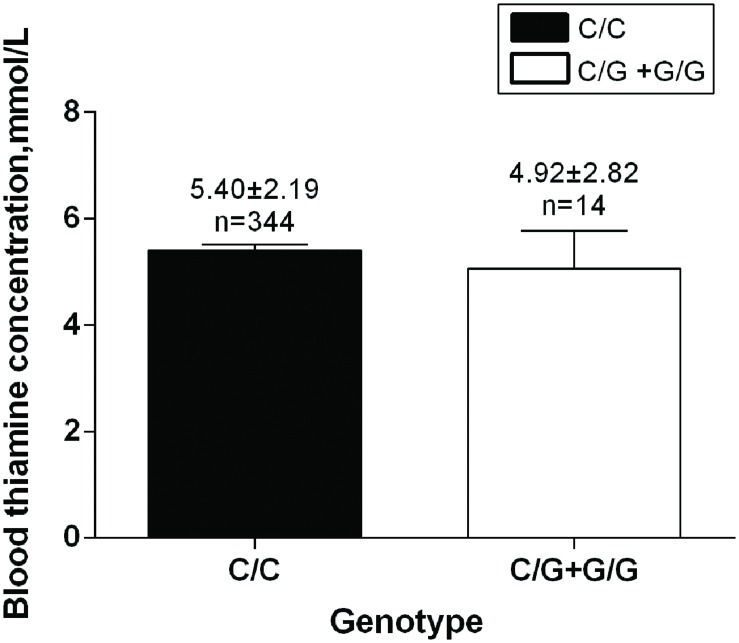
***SLC35F3* genotypes effect on blood thiamine in patients.** C/C genotype: *n* = 344; C/G +G/G genotype: *n* = 14, *p* = 0.551.

No significant difference was detected in Thiamine B1 concentration between rs34032258 genotypes (C/C 5.40 ± 2.19 mmol/L vs. C/G+G/G 4.92 ± 2.82 mmol/L, *p* = 0.551; **Figure 3**).

## Discussion

Zhang et al. (2014) found that rs17514104 and rs16842784 in the *SLC35F3* gene were associated with BP in subjects from North America and Western Europe, through the use of phenotypic extremes and genomic DNA pooling (Zhang et al., 2014). However, we detected neither rs17514104 nor rs16842784 in 93 Chinese people, because allele frequency might be variable in different ethnic population.

We found that rs34032258 was associated with DBP, BUN and Cr, but not with SBP, BMI. This finding was consistent with previous studies showing that DBP but not SBP was substantially heritable (Snieder et al., 2003; Kupper et al., 2005). In addition, in those patients whose DBP ≥ 90 mmHg, G-carriers had higher DBP. This finding indicated that *SLC35F3* may be associated with the regulation of BP and kidney function. We did not observe any association between rs34032258 and BMI. However, we found that there was a lower frequency of G allele of rs34032258 in cases compared with normotensive controls in obesity group. This result suggested that rs34032258 may be involved in obesity-related hypertension.

*SLC35F3* encoded vitamin B1 transporter. According to the prediction using online software^2^, the mutation of *SLC35F3* in rs34032258 (Supplement Figure S1) induced the loose of protein tertiary structure of vitamin B1 transporter, then probably decreased the concentration of vitamin B1 in plasma.

Vitamin B1, a water-soluble vitamin, played an important role in intracellular glucose metabolism. As a coenzyme for α-ketoglutarate-dehydrogenase, it was implicated in the tricarboxylic acid (TCA) cycle, catalyzing the oxidation of ketoglutaric acid to succinyl-CoA. In addition, it was a coenzyme for the pyruvate dehydrogenase complex (PDHC), converting pyruvate to acetyl-CoA. Clinical studies have shown that vitamin B1 might be associated with cardiovascular diseases (Wilkinson et al., 1997). Vitamin B1 supplementation was reported to reduce blood pressure, especial in patients combined with hyperglycemia (Alaei-Shahmiri et al., 2015). In accordance with the result, thiamine repletion could relieve the symptoms of hypertension and hyperinsulinemia in spontaneously hypertensive rats (SHR; Tanaka et al., 2007). However, the underlying mechanisms are still unknown.

One of the mechanisms might be that vitamin B1 could ameliorate the endothelium-dependent vasodilation (Subodh Arora et al., 2006). Routine administration of thiamine might improve endothelial function and therefore slowed the progression of atherosclerosis, especially in patients with impaired glucose tolerance (IGT) who were prone to develop accelerated atherosclerosis (Subodh Arora et al., 2006). The *SLC35F3* mutation could result in the shortage of vitamin B1, then increased the blood pressure.

In addition, vitamin B1 might be related to the cardiac function (Han et al., 1995). Reduction of vitamin B1 was found to be involved the accumulation of intermediate products in glucose metabolism such as pyruvic acid and lactic acid, which could stimulate expansion of peripheral arterial, reduce peripheral resistance, and increase venous return, cardiac output and blood pressure (Zenuk et al., 2003).

The present study may have some limitations. Although our study sample size was considerable, we might not be powerful enough to capture some potential rare but functional causal variants in the *SLC35F3* gene. These disease-causing variants could be in linkage disequilibrium with rs34032258.

In summary, this study is the first to report the association between a missense variant, rs34032258, in the *SLC35F3* gene and hypertension in a Chinese Han population. Further study is needed to discover the molecular mechanisms of *SLC35F3* to blood pressure in order to improve clinical treatment. Our results may have implications for the pathogenesis and treatment of systemic hypertension.

## Author Contributions

All the authors participated in the whole work. However, X-LZ and W-QH took on great work about design of the work and analysis, or interpretation of data. F-PY, K-DJ made contributions to perform the experiment and select samples. GH, S-NW took on drafting the work or revising it critically for important intellectual content. J-GW, P-JG agreed to be accountable for all aspects of the work in ensuring that questions related to the accuracy or integrity of any part of the work are appropriately investigated and resolved.

## Conflict of Interest Statement

The authors declare that the research was conducted in the absence of any commercial or financial relationships that could be construed as a potential conflict of interest.

## Footnotes

1 http://analysis.bio-x.cn/SHEsisMain.htm

2 http://raptorx.uchicago.edu/

## Abbreviations

ANOVA: analysis of variance
BMI: body mass index
BP: blood pressure
BUN: blood urea nitrogen
Cr: creatinine
DBP: diastolic blood pressure
F: female
HR: heart rate
MAF: minor allele frequency
SBP: systolic blood pressure
MANCOVA: multivariate analysis of covariance
SNP: single-nucleotide polymorphism
TC: total cholesterol
TG: triglyceride
WC: waist circumference.
