# Supplementary material for: Association of a SNP in SLC35F3 Gene with the Risk of Hypertension in a Chinese Han Population
Source: Front Genet. 2016 Jun 20;7:108. doi: 10.3389/fgene.2016.00108 (PMC4913099; doi:10.3389/fgene.2016.00108)
Supplement: Supplementary file 1 [file Table_1.PDF]

Supplementary Material

Association of a SNP in *SLC35F3* gene with the risk of hypertension in a Chinese Han Population

Xiao-Li Zang[1.2.3], Wei-Qing Han[1.2.3], Feng-Ping Yang[4], Kai-Da Ji[1.2.3], Ji-Guang Wang[1.2.3], Ping-Jin Gao[1.2.3], Guang He[4*], Sheng-Nan Wu[1.2.3*]

[1] Shanghai Key Laboratory of Vascular Biology at Ruijin Hospital and Shanghai Institute of Hypertension, Shanghai Jiao Tong University School of Medicine, Shanghai, China;

[2] Laboratory of Vascular Biology, Institute of Health Sciences, Shanghai Institutes for Biological Sciences, Chinese Academy of Sciences, Shanghai, China;

[3] State Key Laboratory of Medical Genomics, Shanghai, China;

[4] Bio-X Institutes, Key Laboratory for the Genetics of Developmental and Neuropsychiatric Disorders (Ministry of Education), Shanghai Jiao Tong University, 600 Wan Ping Nan Road, Shanghai 200030, China.

[*]**Correspondence:**

Sheng-Nan Wu: snwu@sibs.ac.cn

Guang He: heguang@sjtu.edu.cn.

2.1 Supplementary Table

Table S1.The number of samples for each analysis

Analytic process	Case	Control	Note
Table1.General characteristics of the study population	1060	1467	
Table2.*SLC35F3* variant rs34032258: Effect on hypertensive traits in case groups	1060	0	
Table3.Allelic frequencies of rs34032258 in different BMI levels	1035	1467	25 BMI data in case groups were missed
Table4.The effect of gender on SBP and DBP	1060	1467	
Figure1.Typical sequences of specific mutations in the *SLC35F3* SNPs in hypertensive patients	93	0	Randomly selected 93 hypertensive patients in order to Sanger sequencing in the first stage of our study.
Figure2.*SLC35F3* genotypes effect on blood thiamine in patients	344	0	Only 344 samples were available for ELISA.
Figure3.DBP level between G-carriers and wild type in those whose DBP≥90 mmHg	578	0	Selected data whose DBP≥90 mmHg in case groups in order to further explore the relation between DBP and rs34032258.

2.2 Supplementary Figure

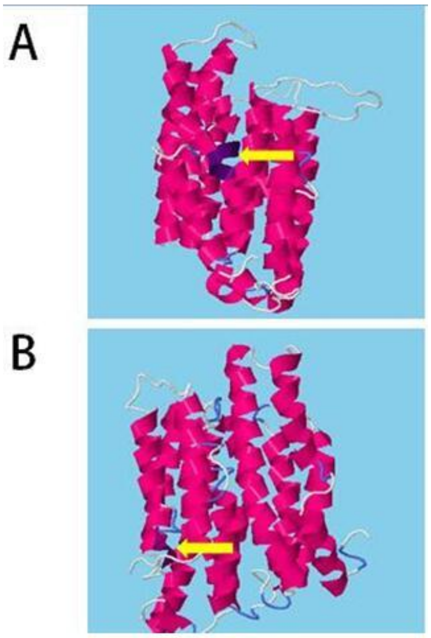

Figure S1. The protein tertiary structure of vitamin B1 transporter predicted by online software
(A) In wild type, the encoded amino acid is arginine (Arg) (http://raptorx.uchicago.edu/StructurePrediction/myjobs/73845858_83920/).
(B) In mutant type, the encoded amino acid is glycine (Gly) and the protein tertiary structure is much looser. (http://raptorx.uchicago.edu/StructurePrediction/myjobs/32552875_83921)
