# Supplementary figures and images for: Association of a SNP in SLC35F3 Gene with the Risk of Hypertension in a Chinese Han Population
Source: Front Genet. 2016 Jun 20;7:108. doi: 10.3389/fgene.2016.00108 (PMC4913099; doi:10.3389/fgene.2016.00108)

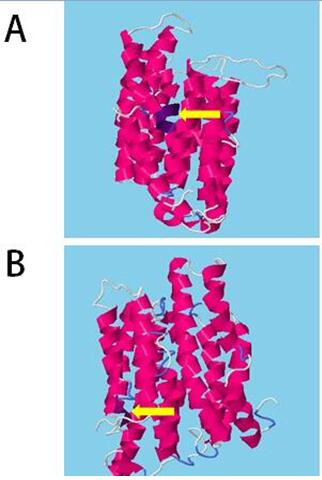

Supplement: Supplementary file 2 [file Image_1.TIF]
